# Supplementary material for: Analysis of how the spatial and temporal patterns of fire and their bioclimatic and anthropogenic drivers vary across the Amazon rainforest in El Niño and non-El Niño years
Source: PeerJ. 2021 Oct 7;9:e12029. doi: 10.7717/peerj.12029 (PMC8502451; doi:10.7717/peerj.12029)
Supplement: Supplemental Information 1 [file peerj-09-12029-s001.docx]

**SUPPLEMENTARY MATERIALS**

**Supplementary 1**: The distribution of land cover types varies across the different countries that fall overlap the Amazon rainforest.


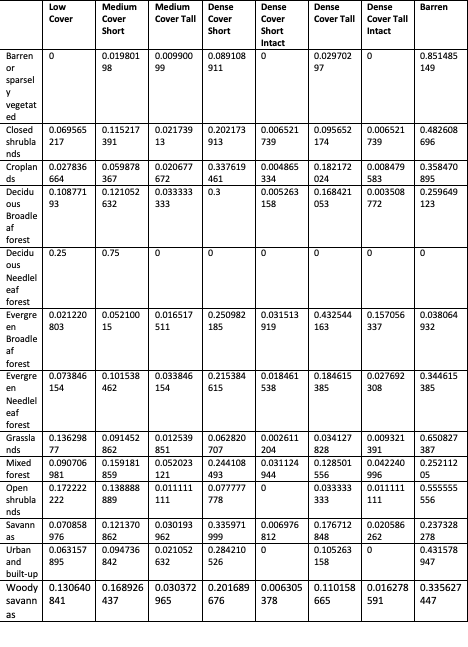


**Supplementary 2: Dry Seasons of El-Nino Years**

**Size:**


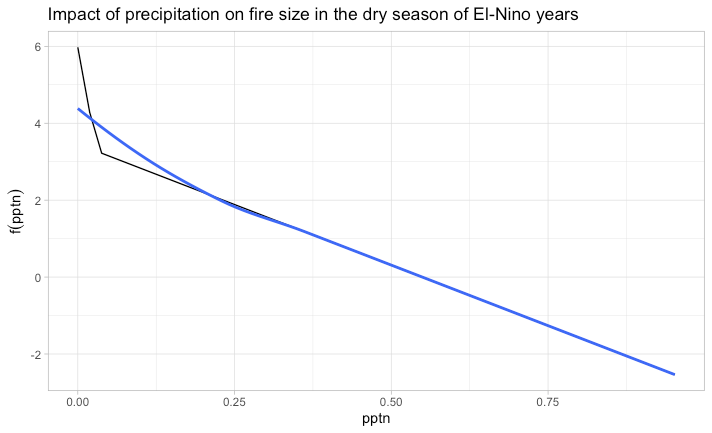


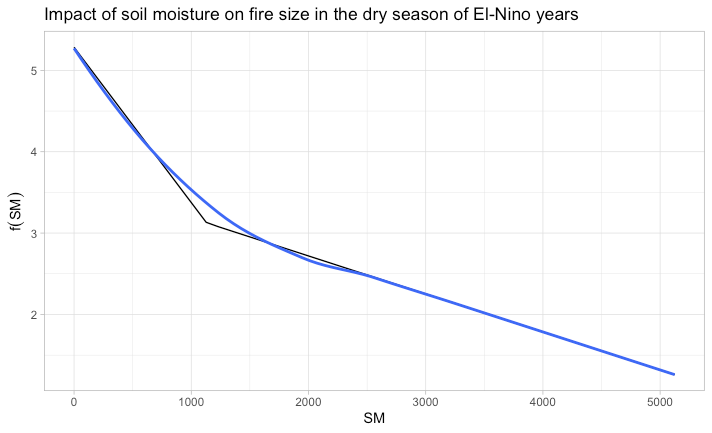


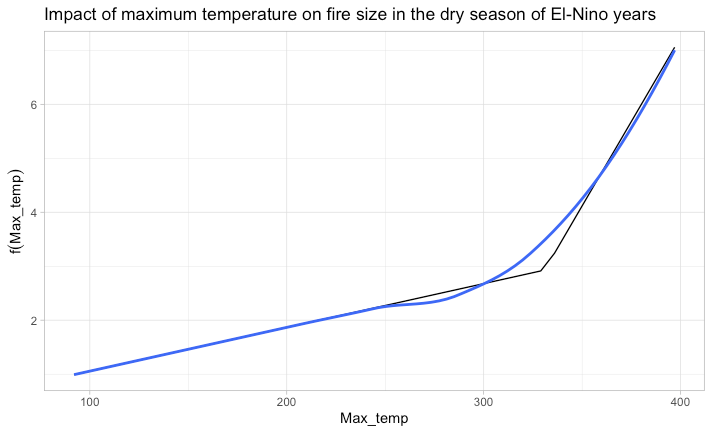


**Speed:**


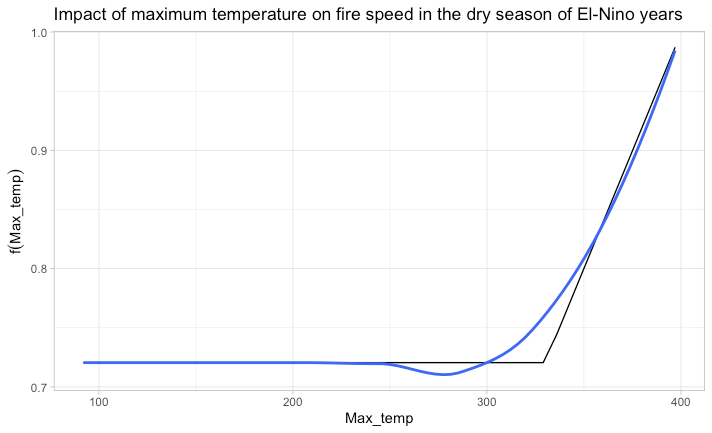


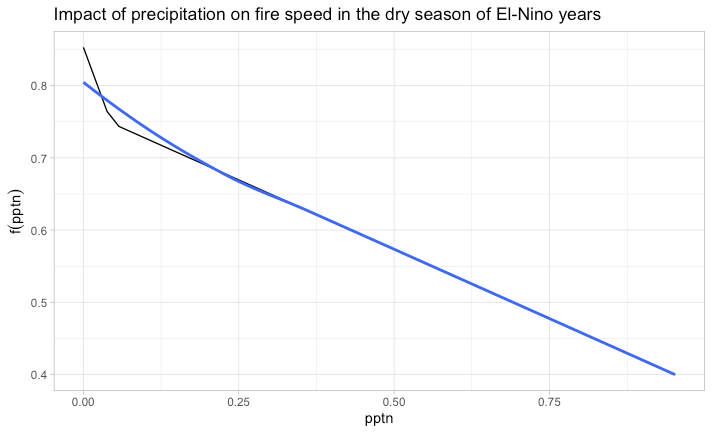


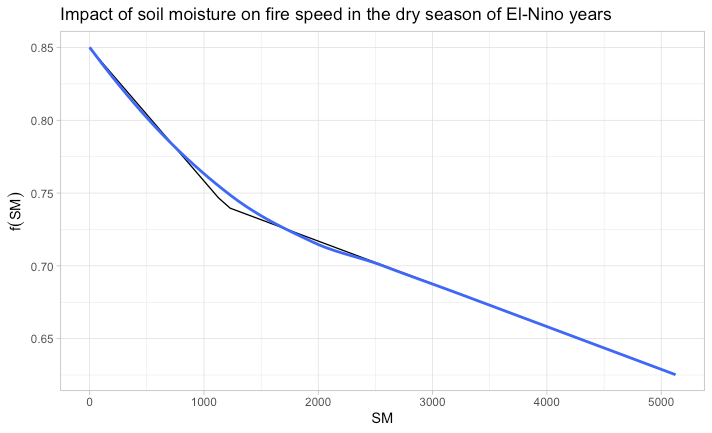


**Duration**


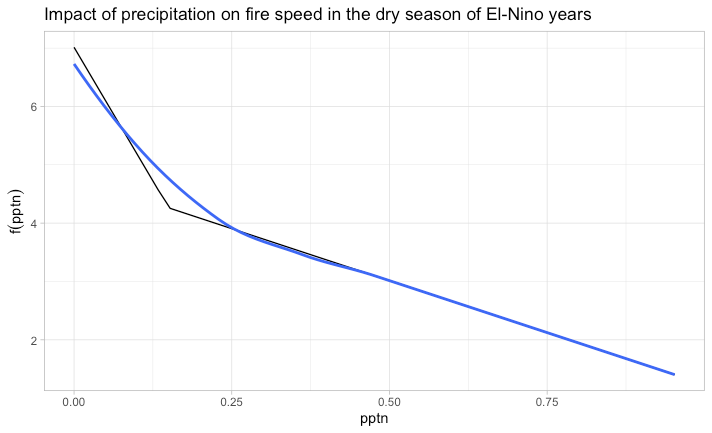


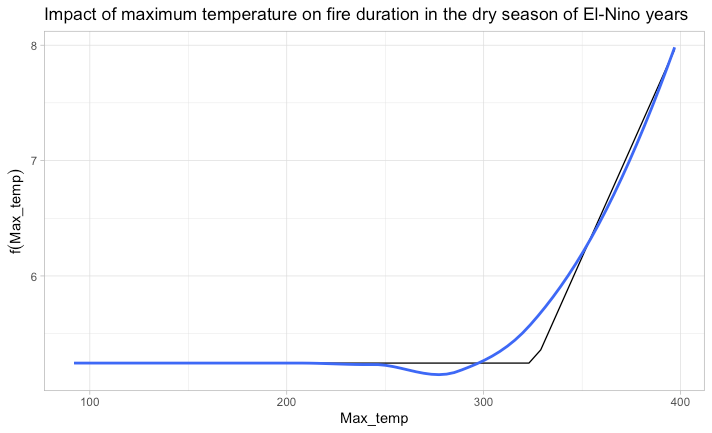


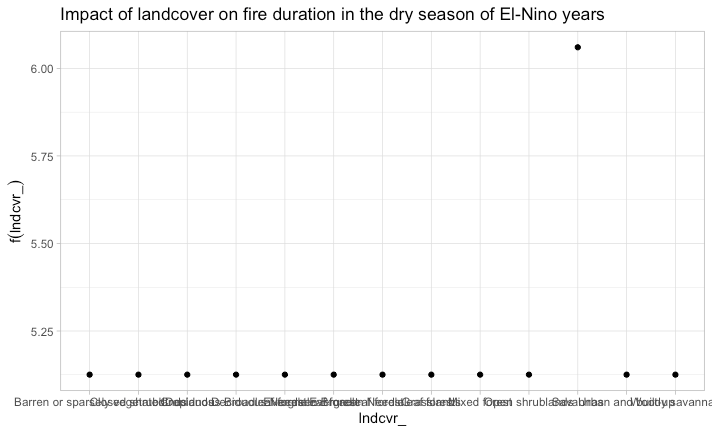


**Expansion**

**
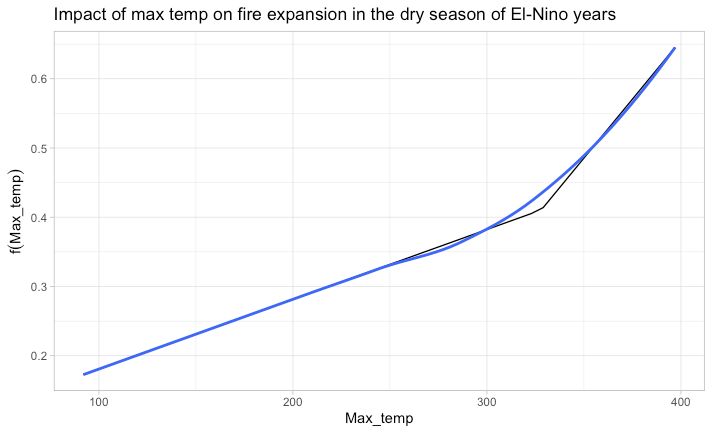
**

**Supplementary 3: Wet Seasons of El-Nino Years**

**Size**

**
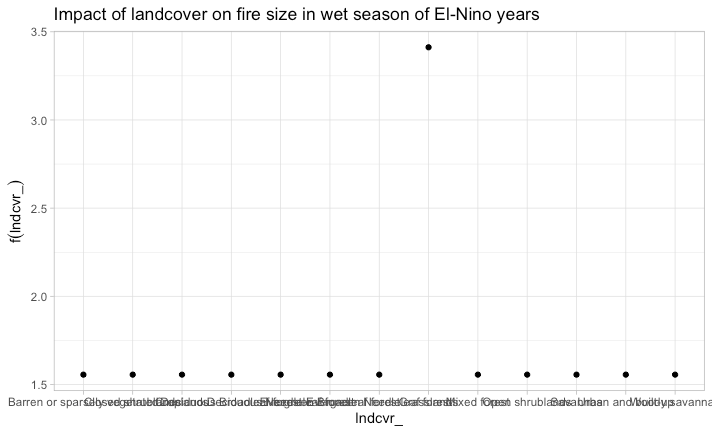
**

**
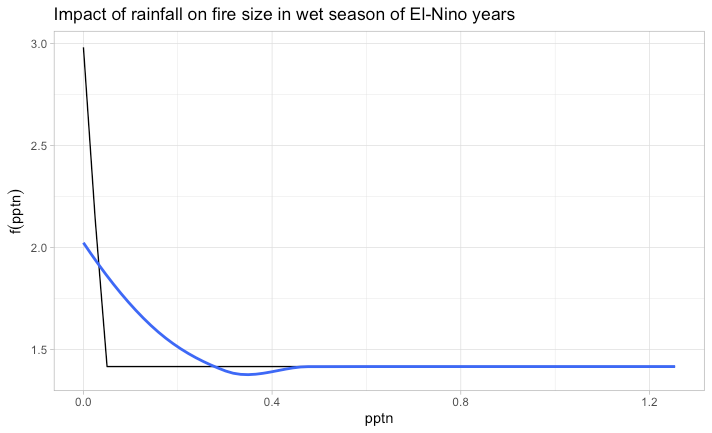
**

**Speed**

**
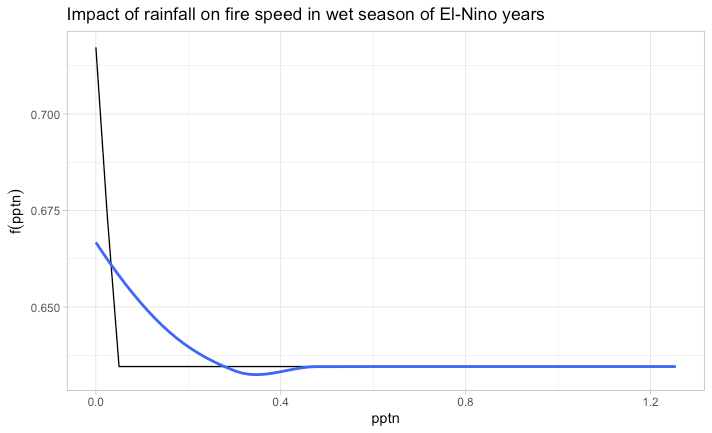
**


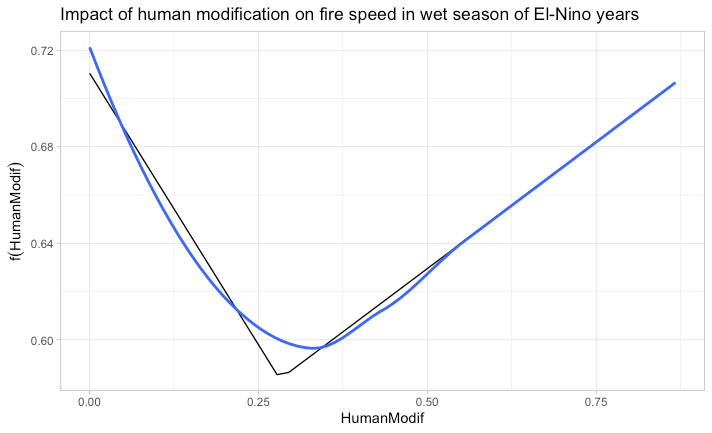


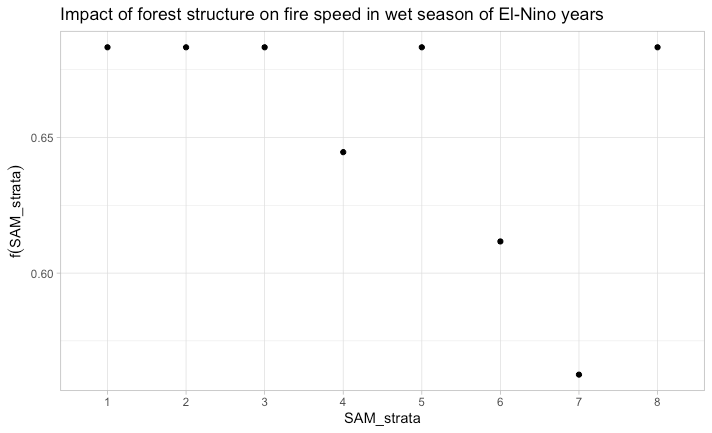


**Duration**


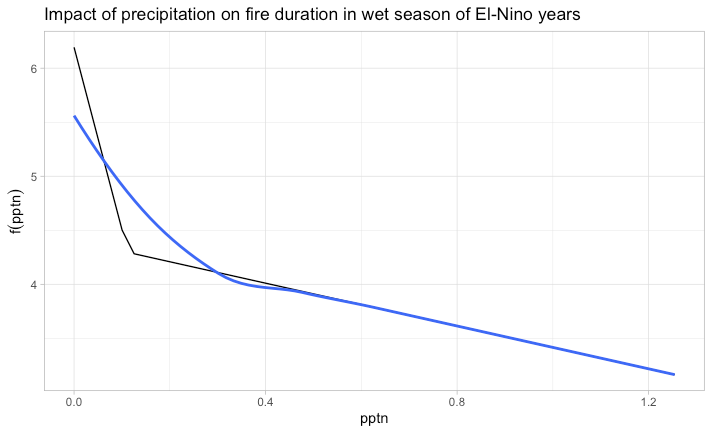


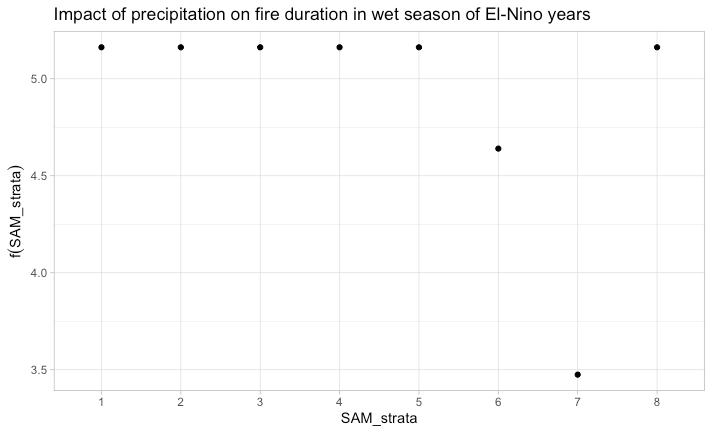


**Expansion**


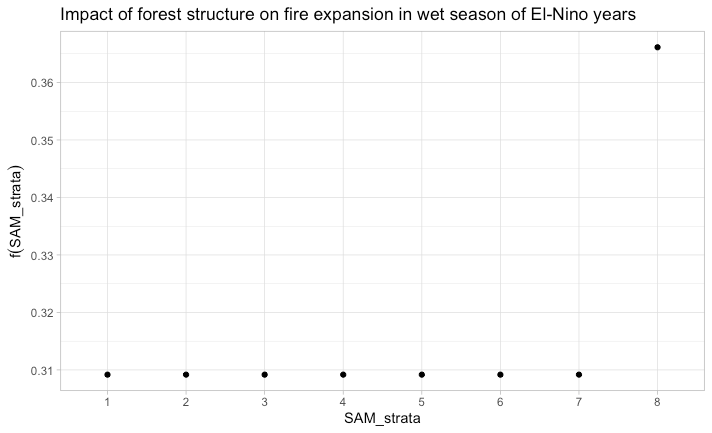


**Supplementary 4:Dry Seasons of Non-El-Nino Years**

**Size**


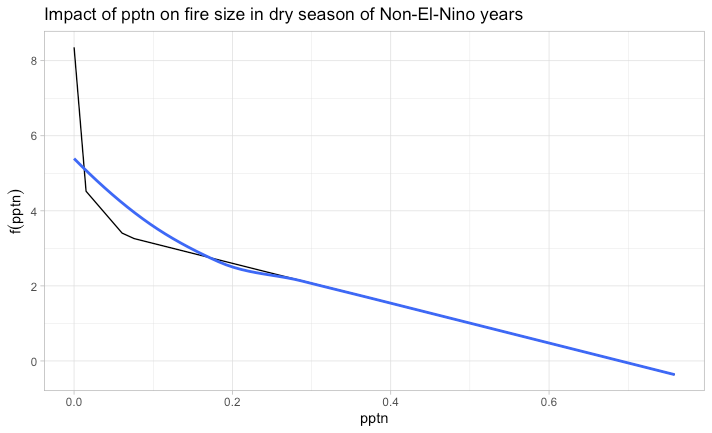


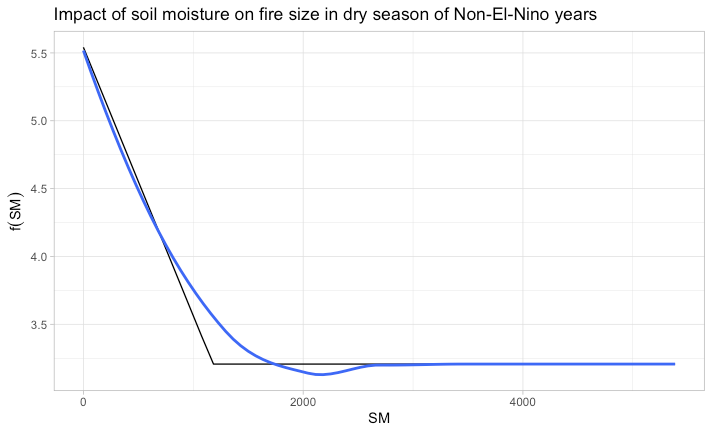


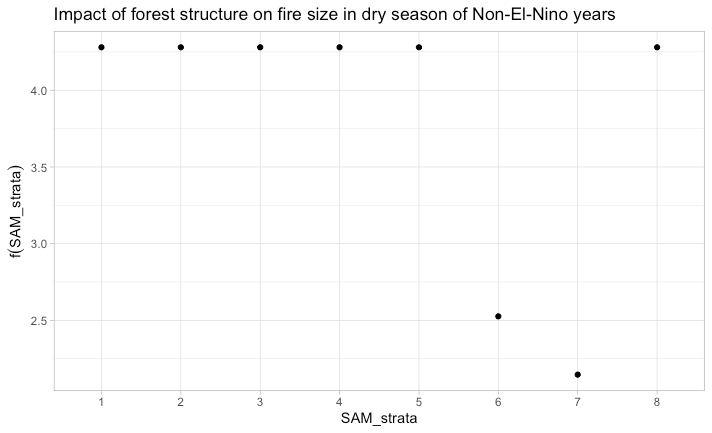


**Speed**:


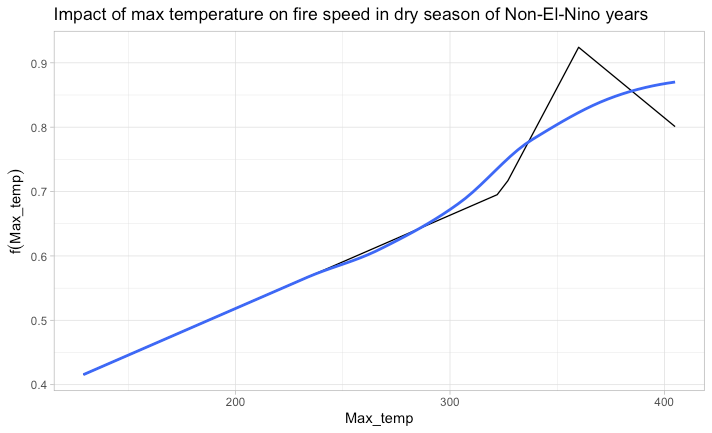


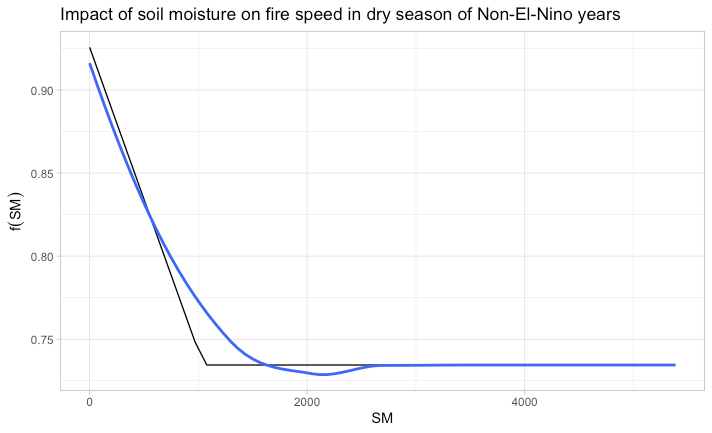


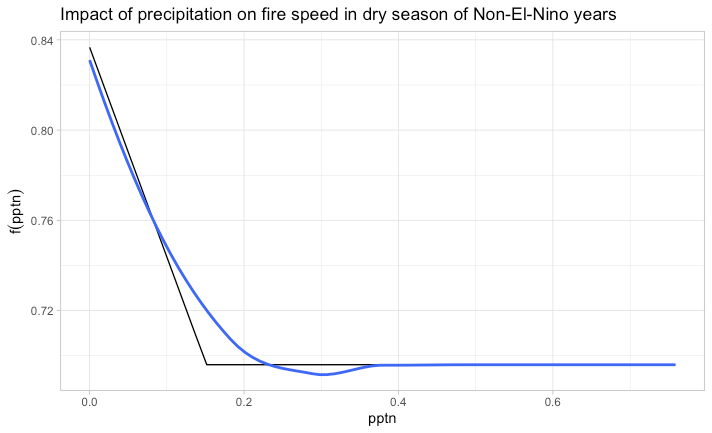


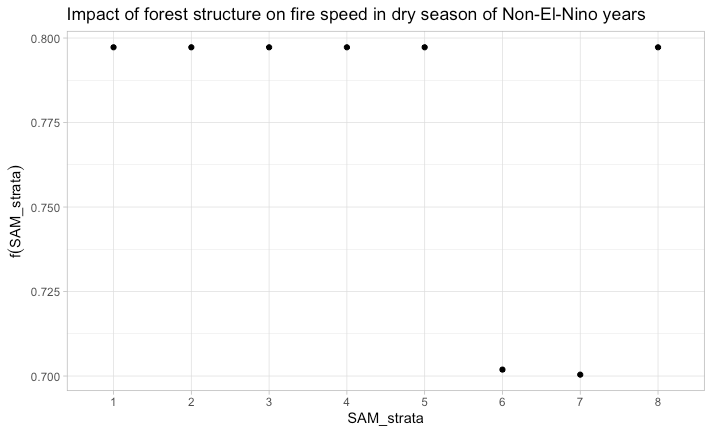


**Duration:**

**
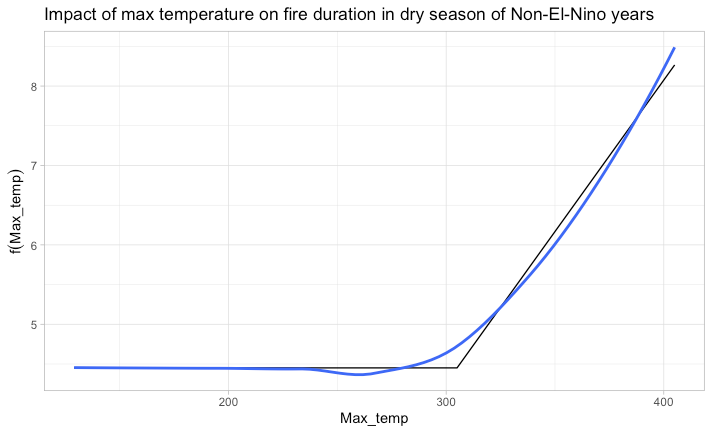
**

**
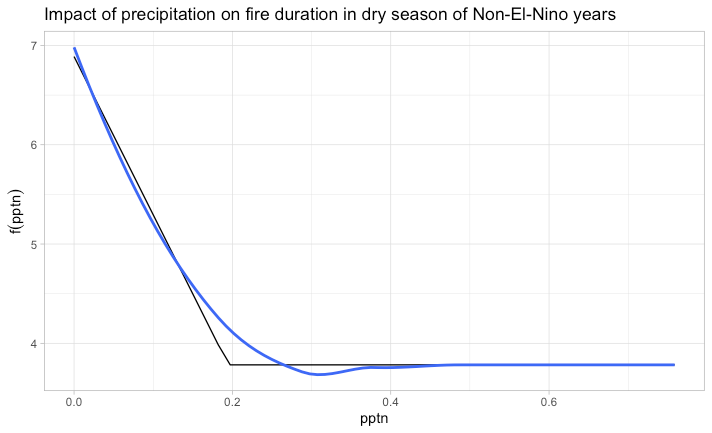
**

**
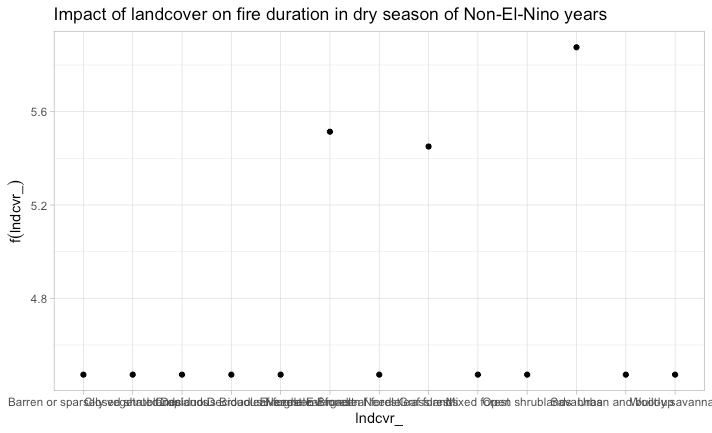
**

**Expansion:**


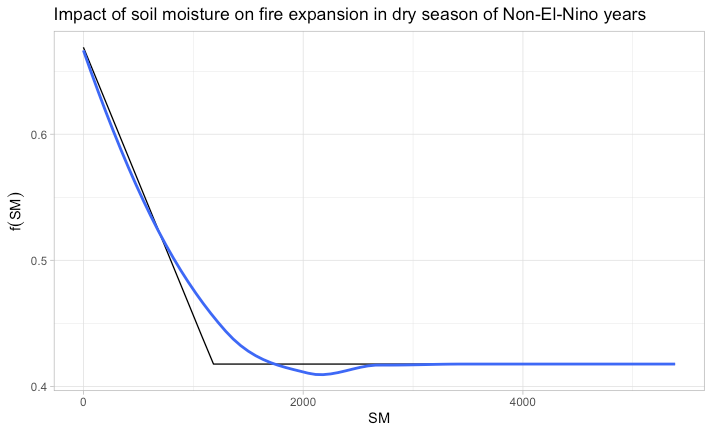


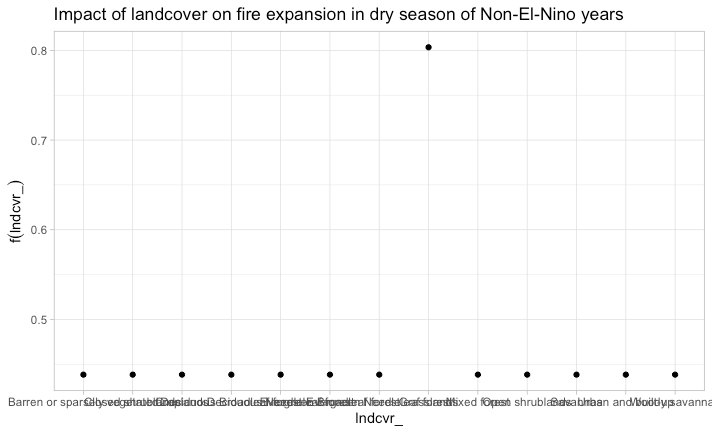


**
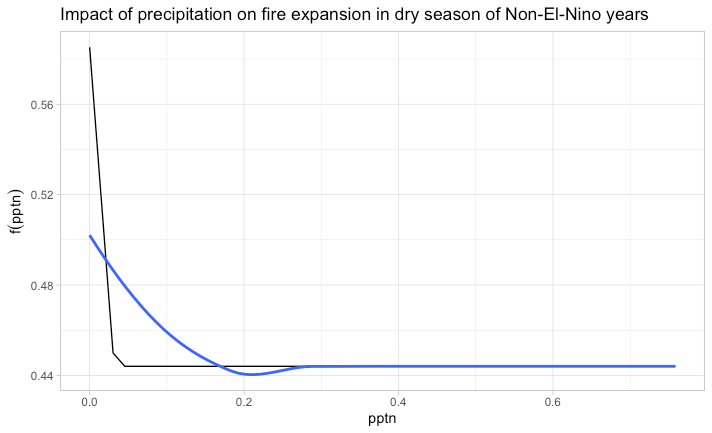
**

**
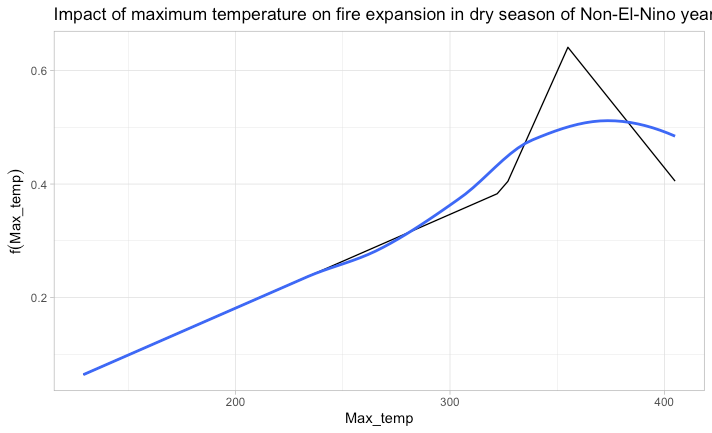
**

**Supplementary 5:Wet Seasons of Non-El-Nino Years**

**Size**

**
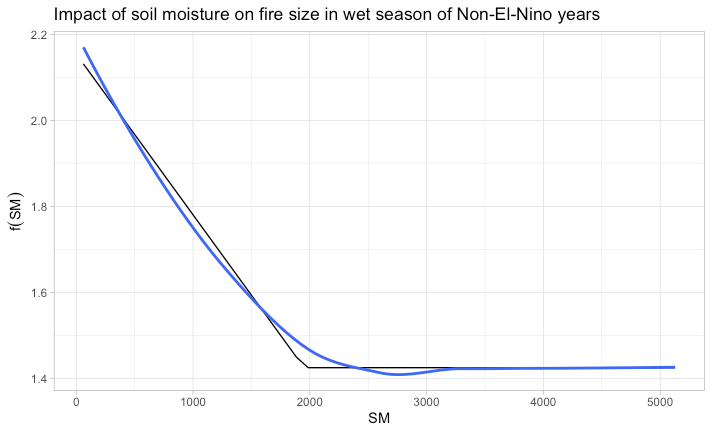
**

**
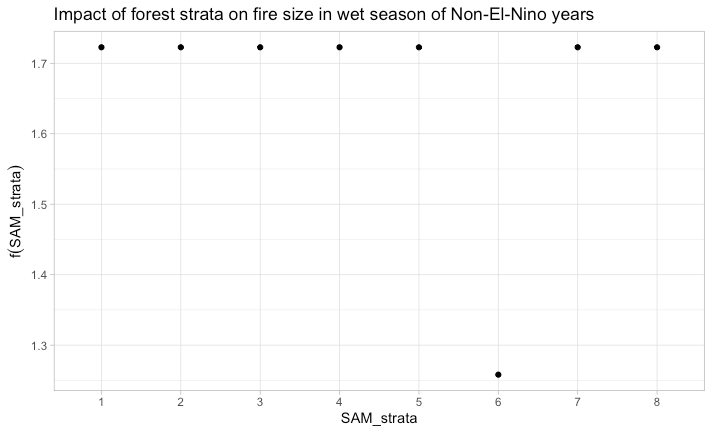
**

**Speed**

**
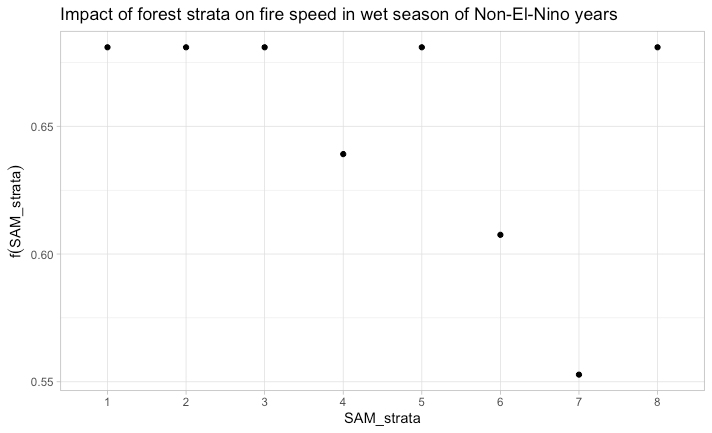
**

**
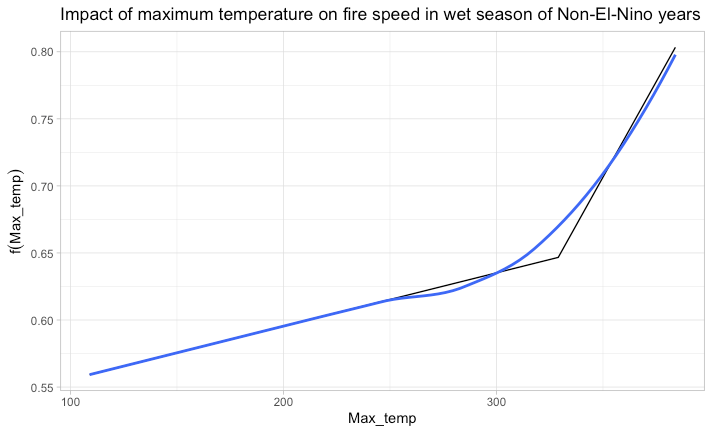
**

**
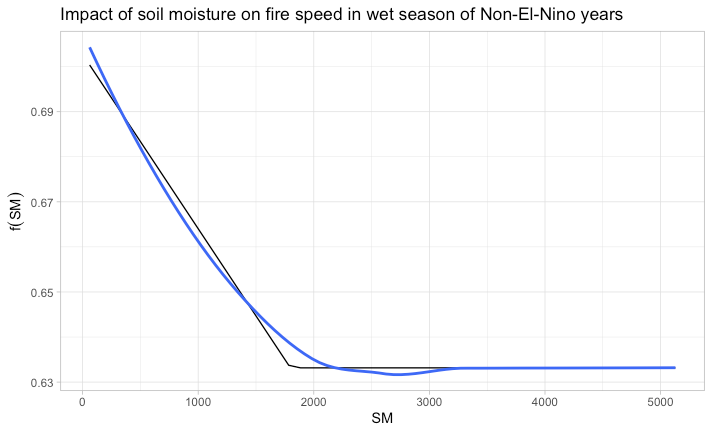
**

**Duration**

**
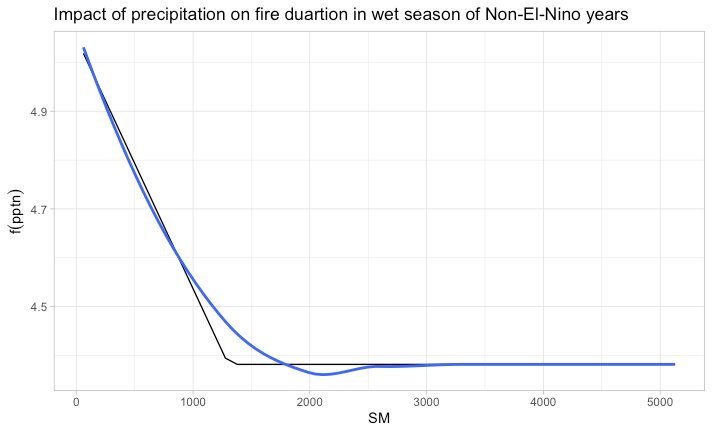
**

**
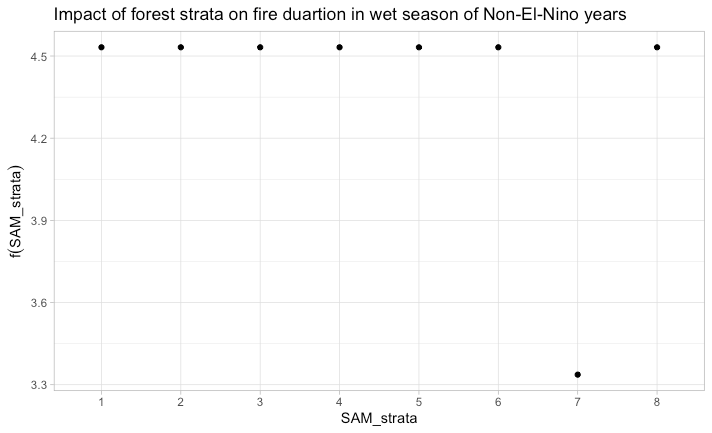
**

**Supplementary 3**

**Country: Bolivia**

| **Year/Month Type** | **Size** | **Dur** | **Perimeter** | **Speed** | **Length** | **PDSI** | **SM** | **Rainfall** | **Temperature** |
| --- | --- | --- | --- | --- | --- | --- | --- | --- | --- |
| El Nino-Dry | 5.48932 | 6.05131 | 10.03448 | 0.943355 | 1.195229 | 100.6775 | 1147.417 | 0.074803 | 321.1433639 |
| El Nino-Wet | 1.99549 | 4.275346 | 6.000178 | 0.729438 | 0.872221 | 88.07304 | 1587.816 | 0.135528 | 316.1907647 |
| Non El Nino-Dry | 5.147771 | 5.561177 | 9.49861 | 0.92707 | 1.219286 | 116.5053 | 1050.543 | 0.080378 | 327.3235566 |
| Non El Nino-Wet | 1.625571 | 3.818397 | 5.318338 | 0.685446 | 0.818584 | 213.2639 | 1705.307 | 0.168103 | 312.4998818 |

**Country: Brazil**

| **Year/Month Type** | **Size** | **Dur** | **Perimeter** | **Speed** | **Length** | **PDSI** | **SM** | **Rainfall** | **Temperature** |
| --- | --- | --- | --- | --- | --- | --- | --- | --- | --- |
| El Nino-Dry | 2.601787 | 5.543062 | 6.386248241 | 0.686391 | 0.718526 | -56.9476 | 1383.903 | 0.090186 | 334.5127171 |
| El Nino-Wet | 1.608582 | 5.142511 | 5.305489731 | 0.632629 | 0.620718 | -97.2232 | 1790.776 | 0.123435 | 324.7588125 |
| Non El Nino-Dry | 3.326945 | 5.674811 | 7.143936573 | 0.71772 | 0.802342 | -92.3888 | 1343.503 | 0.094528 | 336.4492187 |
| Non El Nino-Wet | 1.44407 | 4.432285 | 4.962419026 | 0.63092 | 0.656514 | 3.33867 | 2090.002 | 0.154343 | 321.9133082 |

**Country: Colombia**

| **Year/Month Type** | **Size** | **Dur** | **Perimeter** | **Speed** | **Length** | **PDSI** | **SM** | **Rainfall** | **Temperature** |
| --- | --- | --- | --- | --- | --- | --- | --- | --- | --- |
| El Nino-Dry | 1.597149 | 3.813861 | 5.392 | 0.691861 | 0.822772 | 182.5723 | 1757.255 | 0.352676 | 311.6475248 |
| El Nino-Wet | 2.329059 | 4.812528 | 6.115260254 | 0.687428 | 0.756121 | 160.3693 | 969.54 | 0.136021 | 319.2918409 |
| Non El Nino-Dry | 1.209545 | 3.531469 | 4.467132867 | 0.625874 | 0.703881 | 195.3776 | 1632.629 | 0.360363 | 305.8356643 |
| Non El Nino-Wet | 2.081393 | 4.877258 | 5.935403428 | 0.683661 | 0.731389 | 124.2729 | 740.8734 | 0.123002 | 323.0009264 |

**Country: Ecuador**

| **Year/Month Type** | **Size** | **Dur** | **Perimeter** | **Speed** | **Length** | **PDSI** | **SM** | **Rainfall** | **Temperature** |
| --- | --- | --- | --- | --- | --- | --- | --- | --- | --- |
| El Nino-Dry | 0.461667 | 2.333333 | 2.6225 | 0.500833 | 0.558333 | 224.1667 | 585.8333 | 0.143436 | 279.9166667 |
| El Nino-Wet | 0.593415 | 3.04878 | 3.093414634 | 0.52122 | 0.490244 | -78.3171 | 559.2927 | 0.234565 | 292.4634146 |
| Non El Nino-Dry | 0.32 | 1 | 2.315 | 0.46 | 0.695 | 440 | 994 | 0.167382 | 240.5 |
| Non El Nino-Wet | 0.425 | 1.333333 | 2.621666667 | 0.471667 | 0.666667 | 188.6667 | 361.5 | 0.309585 | 295.8333333 |

**Country: Guyana**

| **Year/Month Type** | **Size** | **Dur** | **Perimeter** | **Speed** | **Length** | **PDSI** | **SM** | **Rainfall** | **Temperature** |
| --- | --- | --- | --- | --- | --- | --- | --- | --- | --- |
| El Nino-Dry | 2.124224 | 5.380172 | 6.353586207 | 0.722052 | 0.749543 | -114.578 | 1164.092 | 0.08125 | 328.8801724 |
| El Nino-Wet | 1.742115 | 5.009443 | 5.784787535 | 0.669268 | 0.69042 | -84.1478 | 1168.86 | 0.078275 | 322.5042493 |
| Non El Nino-Dry | 2.085918 | 5.464419 | 6.349538077 | 0.711798 | 0.724157 | -35.2684 | 1330.03 | 0.087209 | 327.1922597 |
| Non El Nino-Wet | 1.828089 | 4.941879 | 5.743224522 | 0.691911 | 0.731186 | 68.03662 | 1519.067 | 0.075938 | 321.7507962 |

**Country: Peru**

| **Year/Month Type** | **Size** | **Dur** | **Perimeter** | **Speed** | **Length** | **PDSI** | **SM** | **Rainfall** | **Temperature** |
| --- | --- | --- | --- | --- | --- | --- | --- | --- | --- |
| El Nino-Dry | 0.962126 | 4.03938 | 4.025490504 | 0.562692 | 0.531035 | -184.082 | 1296.113 | 0.126984 | 315.0512284 |
| El Nino-Wet | 0.955159 | 4.072464 | 4.019594203 | 0.568058 | 0.585623 | -27.887 | 1192.722 | 0.139079 | 301.7652174 |
| Non El Nino-Dry | 0.888247 | 4.107849 | 3.941174478 | 0.560466 | 0.516872 | -90.4763 | 1416.231 | 0.127319 | 319.6166008 |
| Non El Nino-Wet | 1.384846 | 4.138462 | 4.856692308 | 0.663769 | 0.690615 | -64.7923 | 1222.331 | 0.145608 | 290.0769231 |

**Country: Venezuela**

| **Year/Month Type** | **Size** | **Dur** | **Perimeter** | **Speed** | **Length** | **PDSI** | **SM** | **Rainfall** | **Temperature** |
| --- | --- | --- | --- | --- | --- | --- | --- | --- | --- |
| El Nino-Dry | 1.896259 | 5.42963 | 5.685296296 | 0.681667 | 0.660815 | -124.793 | 1273.741 | 0.200466 | 296.9074074 |
| El Nino-Wet | 1.809393 | 4.966438 | 5.639342229 | 0.669283 | 0.692335 | -203.016 | 1103.968 | 0.059957 | 323.9986521 |
| Non El Nino-Dry | 1.661006 | 4.39645 | 5.188106509 | 0.630414 | 0.762367 | -68.432 | 1356.325 | 0.244173 | 286.6035503 |
| Non El Nino-Wet | 1.880659 | 4.655193 | 5.671279788 | 0.679297 | 0.75436 | -5.75964 | 1309.835 | 0.079058 | 319.0050015 |
